# Novel and innovative resuscitation systems in Japan

**DOI:** 10.1016/j.resplu.2023.100541

**Published:** 2023-12-30

**Authors:** Yohei Okada, Kensuke Fujita, Takayuki Ogura, Tomokazu Motomura, Yuita Fukuyama, Yuki Banshotani, Rina Tokuda, Shinichi Ijuin, Akihiko Inoue, Haruka Takahashi, Shoji Yokobori

**Affiliations:** a Health Services and Systems Research, Duke-NUS Medical School, Singapore; b Department of Preventive Services, Kyoto University, Kyoto, Japan; c Department of Emergency Medicine and Critical Care Medicine, Tochigi Prefectural Emergency and Critical Care Center, Imperial Gift Foundation Saiseikai Utsunomiya Hospital, Japan; d Shock and Trauma Center/Hokusoh HEMS Nippon Medical School Chiba Hokusoh Hospital, Chiba, Japan; e Tajima Emergency and Critical Care Medical Center, Hyogo, Japan; f Department of Emergency and Critical Care Medicine, Hyogo Emergency Medical Center, Hyogo, Japan; g Medical Science, Nippon Sport Science University, Tokyo, Japan; h Department of Emergency and Critical Care Medicine Graduate School of Nippon Medical School, Tokyo, Japan

**Keywords:** Prehospital ECPR, Physician-staffed ambulance, Physician-staffed helicopter, Hybrid ER, Resuscitation, Cardiac arrest

## Abstract

**Aim:**

Out-of-hospital cardiac arrest (OHCA) is a life-threatening emergency that requires rapid and efficient intervention. Recently, several novel approaches have emerged and have been incorporated into resuscitation systems in some local areas of Japan. This review describes innovative resuscitation systems and highlights their strengths.

**Main text:**

First, we discuss the deployment of a physician-staffed ambulance, in which emergency physicians offer advanced resuscitation to patients with OHCA on site. In addition, we describe the experimental practice of extracorporeal membrane oxygenation (ECPR) in a prehospital setting. Second, we describe a physician-staffed helicopter, wherein a medical team provides advanced resuscitation at the scene. We also explain their initiative to provide early ECPR, even in remote areas. Finally, we provide an overview of the “hybrid ER” system which is a “one-fits-all” resuscitation bay equipped with computed tomography and fluoroscopy equipment. This system is expected to help swiftly identify and rule out irreversible causes of cardiac arrest, such as massive subarachnoid hemorrhage, and implement ECPR without delay.

**Conclusion:**

Although these revolutionary approaches may improve the outcomes of patients with OHCA, evidence of their effectiveness remains limited. In addition, it is crucial to ensure cost-effectiveness and sustainability. We will continue to work diligently to assess the effectiveness of these systems and focus on the development of cost-effective and sustainable systems.

## Introduction

Out-of-hospital cardiac arrest (OHCA) is a critical medical emergency with a high mortality rate. Enhancing OHCA patient outcomes rely on efficient implementation of “the chain of survival,” which involves a coordinated series of actions. Significant efforts have been made to improve OHCA outcomes through public education, dispatcher-assisted bystander CPR, high-quality CPR training, widespread use of automated external defibrillators in public places, implementation of extracorporeal CPR (ECPR), and standardized high-quality in-hospital care.^1^, ^2^, ^3^, ^4^, ^5^, ^6^, ^7^ These initiatives led to improvements in patient outcomes in Japan. For example, in Japan, out of the annual total of 120,000 OHCA cases, approximately 25,000 cases witnessed OHCA cases due to presumed cardiogenic causes are reported. The frequency of bystander CPR by citizen volunteers was 48% in 2007 and improved to 57% in 2017. Furthermore, among them, the survival and favorable neurological outcomes were, respectively, 10.2% and 6.1% in 2007, which improved to 13.5% and 8.7% in 2017.^7^ However, the proportion of favorable outcomes has recently plateaued, and they remained at the same level in 2019 (13.9% and 9.0%).^7^, ^8^ To overcome the current stagnation and further improve OHCA outcomes, a revolutionary and novel approach is required. Additionally, the establishment of these innovations as local emergency medical systems is vital for addressing the challenges posed by OHCA and ensuring better patient survival rates.

**Fig. 1:**
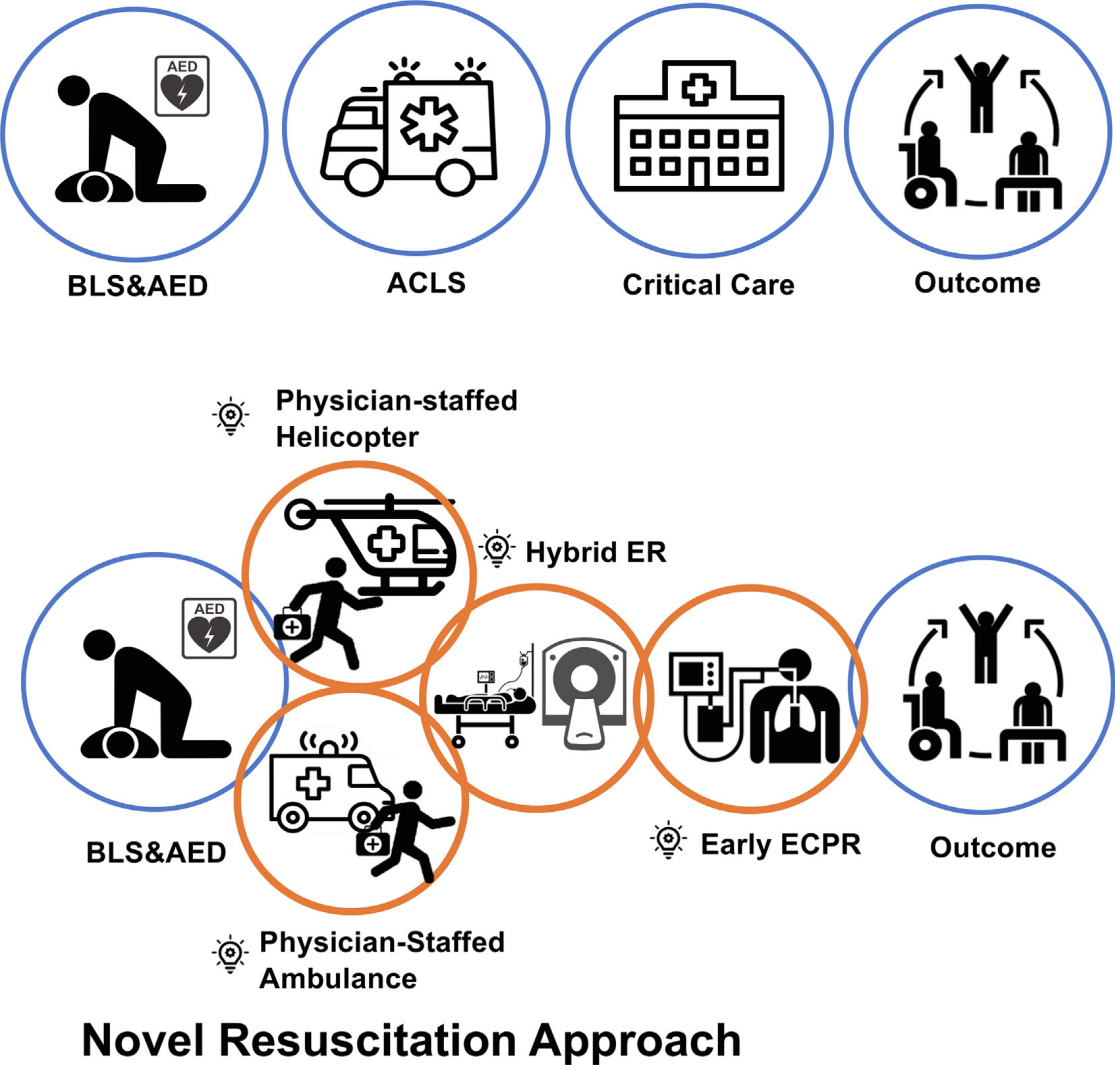
**Concept of the Novel and Innovative Resuscitation Systems in Resuscitation in Japan.** Innovative approaches to resuscitation in Japan. Physician-staffed ambulances and helicopters can provide advanced resuscitation by emergency physicians, and they can also lead to the early implementation of ECPR. Further, the novel strategy using hybrid ER can provide ECPR promptly and safely. They are expected to improve the outcome of cardiac arrest patients.

Recently, several novel approaches have been proposed, including the establishment of resuscitation systems in certain areas of Japan (Fig. 1). One approach involves the use of physician-staffed ambulances and helicopter systems.^9^, ^10^ These systems enable emergency physicians and nurses to provide resuscitation and transfer patients to tertiary care hospitals even when OHCA occurs in rural areas with limited access.^11^, ^12^ Furthermore, these set-ups can be integrated with early hospital ECPR initiatives. As an experimental program, some areas have started implementing prehospital ECPR.^13^ Another notable system is the “hybrid ER,” a resuscitation bay equipped with computed tomography (CT) and fluoroscopy equipment, which enables the clinicians to induce ECPR and detect the potential cause to be treated.^14^ Although the evidence of the effectiveness of these systems has not been fully investigated, we believe that these novel systems have the potential to improve the outcomes of OHCA patients. This review describes these systems in Japan, summarizes relevant evidence, and provides an overview of their strengths and prospects.

## Physician-Staffed Ambulance, “Doctor-Car,” system in Japan

In Japan, emergency medical services (EMS) provide standardized prehospital emergency care. The EMS operates as part of the local fire department overseen by the Fire and Disaster Management Agency under the Ministry of Internal Affairs and Communication. Each ambulance in the EMS system is staffed by three paramedics with at least one certified emergency life-saving technician, which is comparable to emergency medical technicians in other countries.^7^ They are trained for prehospital emergency care and authorized for certain medical procedures such as inducing tracheal intubation or supraglottic airway devices, administering adrenaline to cardiac arrest patients, and providing intravenous crystalloids to patients suspected of having hypovolemic shock and glucose to patients with hypoglycemia.^7^ However, compared with Western countries, Japanese law imposes significant limitations on the scope of EMS activities.^7^, ^9^ For instance, EMS paramedics in Japan are not permitted to use other resuscitative drugs (such as amiodarone hydrochloride, lidocaine, and magnesium sulfate) for advanced resuscitation.^7^ Given the context in Japan, to improve the outcomes of OHCA patients, the concept of a physician-staffed ambulance, known in Japan as “Doctor-Car,” has garnered considerable attention within the field of prehospital emergency care.^9^

The physician-staffed ambulance system in Japan is defined as an “emergency vehicle equipped with essential medical equipment and medications required for delivering medical treatment outside the hospital environment, and staffed by a qualified physician.” Their purposes are to respond to incidents or disasters, provide emergency care to severely injured or critically ill individuals, and facilitate interfacial medical transfers.^9^ Japan's physician-staffed ambulance system closely resembles France's “Service d’Aide Médicale Urgente (SAMU)”.^15^ Upon dialing the emergency number (119) which is equivalent to 911, dispatchers identify the cases suspected of being severe based on the provided information and promptly dispatch a physician-staffed ambulance affiliated with medical institutions in the respective region. In Japan, drawing inspiration from various international models, the physician-staffed ambulance system has been gradually introduced since the early 1990s as part of an initiative by the Ministry of Health, Labour, and Welfare.

**Fig. 2:**
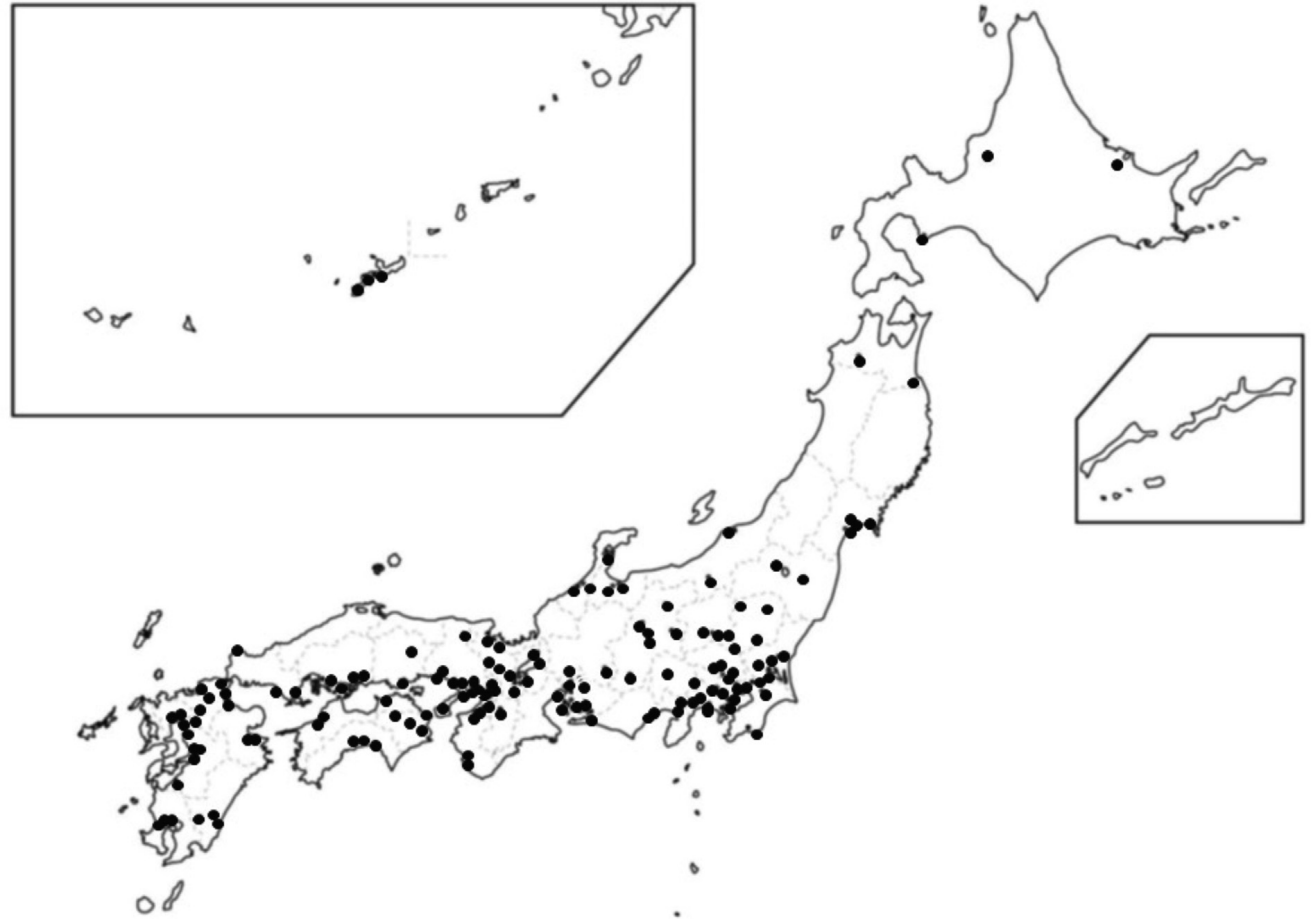
**Distribution of Physician-Staffed Ambulances in Japan.** The dots on the map represent the locations of emergency medical centers implementing the physician-staffed ambulance system in 2018. In 2019, 216 physician-staffed ambulance systems were in operation.

In 2018, Igarashi et al. presented the nationwide distribution of the physician-staffed ambulance system (Fig. 2).^9^ The number of physician-staffed ambulances operated by tertiary emergency medical centers and the number of annual dispatches showed an increasing trend until 2017. By 2019, 216 physician-staffed ambulance systems were operational, with 29,271 dispatches annually.^16^

However, challenges such as staffing and operational costs have constrained the availability of facilities that operate physician-staffed ambulances.

Although numerous studies have explored the efficacy of physician-staffed ambulances, comprehensive nationwide research on this subject in Japan is limited. Hagihara et al. used nationwide OHCA registry data from 2005 to 2010 and found that the presence of a physician was associated with improved outcomes, such as return of spontaneous circulation before hospital arrival, 1-month survival, and good neurological outcomes. Similarly, Sato et al. indicated that physician-staffed ambulances were associated with favorable neurological outcomes in patients with OHCA.^12^, ^17^ Although this study demonstrated the potentially favorable impact of physician-staffed ambulances on patients with OHCA, it has some limitations, such as the residual potential confounding of unmeasured baseline characteristics. Therefore, further investigations using high-quality prospective data are necessary to clarify the effectiveness of physician-staffed ambulance systems.

## Physician-staffed ambulance and prehospital ECPR program

We provide an overview of the physician-staffed ambulance system that provides prehospital ECPR in Utsunomiya City, which facilitates prehospital ECPR. This method of ECPR delivery was first reported in Paris in 2012.^18^ It is practiced in limited regions in France, Germany, and the Netherlands, and in some cities in the US (Minnesota and New Mexico).^19^, ^20^, ^21^, ^22^, ^23^ In 2016, the first case of prehospital ECPR in Japan was reported; since then, the development of a system to provide prehospital ECPR has been discussed. To date, no other Asian country has reported their implementation.^24^

Japan has one center offering ECPR in a pre-hospital setting located in Utsunomiya City, 100 km from Tokyo. This hospital provides tertiary emergency care and performs approximately 100 ECPR cases annually, and it serves a region with a population of approximately 600,000, including neighboring areas. Since 2020, it has been operating on weekdays, covering the entire city of Utsunomiya and its surrounding regions, regardless of distance. However, it still has limited coverage compared to the SAMU in Paris which covers 2.2 million residents in Paris and 12 million people in the influence area, or the Minnesota mobile ECMO program in the Minneapolis–Saint Paul metropolitan area with 3.7 million residents.^15^, ^23^

The inclusion criteria for prehospital ECPR were as follows: 75 years old or younger, witnessed or estimated no-flow time <10 min, bystander CPR provided, initial rhythm as shockable or PEA, and pH value in blood gas assessment >7.0. Occasionally, it is challenging to efficiently gather clinical information in a prehospital setting and make appropriate patient selections. Consequently, this team considered the presence of signs of life as a more crucial criterion for evaluating prehospital ECPR candidates than the initial cardiac rhythm, estimated no-flow time, or SAMU.^25^

**Fig. 3:**
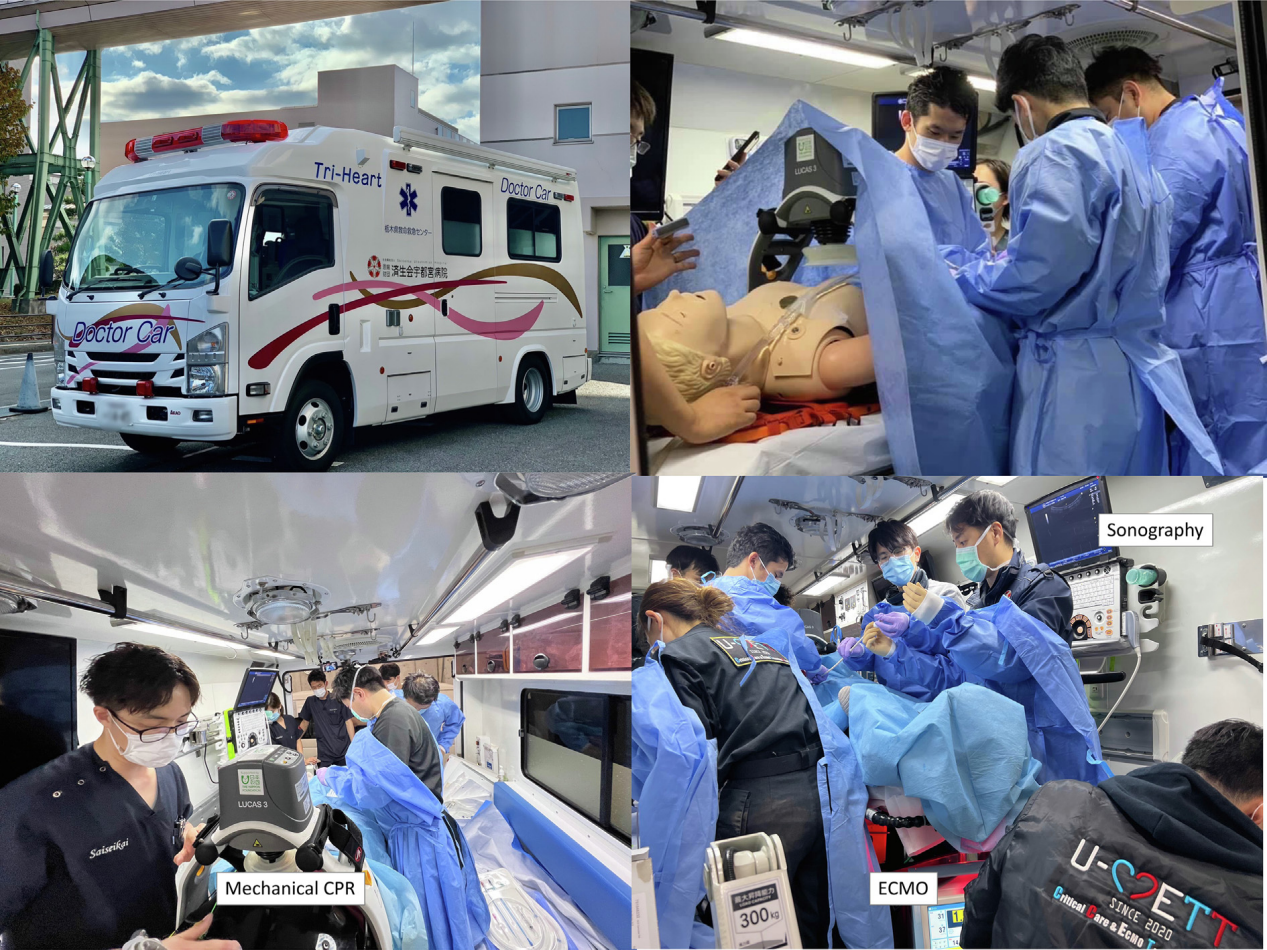
**Prehospital ECPR in Utsunomiya City.** These pictures are of the physician-staffed Ambulance to provide prehospital ECPR and simulation training for providing prehospital ECPR. While providing mechanical CPR, cannulation is performed under sonography guidance by the emergency physician. The prehospital cannulation team includes two emergency physicians, one nurse, and emergency medical technicians. ECPR: Extracorporeal cardiopulmonary resuscitation.

The physician-staffed ambulance for prehospital ECPR called the “Mobile ICU” has a spacious area and ample supplies such as power and gas, and is equipped with a blood gas analyzer, sonography, refrigerator for blood product storage, an ECMO gas blender, and a heater-cooler unit (Fig. 3). During prehospital ECPR, procedures can be performed in a manner similar to those performed in an emergency department. The team aboard the ambulance included two emergency physicians, one nurse, and two emergency medical technicians affiliated with the hospital. They were trained in ECMO management, in addition to resuscitation. They are dispatched by local dispatch centers when a suitable candidate arises.

One of the advantages of the prehospital ECPR system is its potential to shorten the time required to initiate ECPR while ensuring high-quality CPR at the scene by eliminating the need for transfer to the hospital. Generally, cardiac arrest is a time-critical condition and any delay in ECPR initiation can lead to worse outcomes. Therefore, many regions in Japan have adopted protocols in which paramedics prioritize transferring ECPR candidates to hospitals rather than providing intubation or administering adrenaline at the scene to minimize the time required to initiate ECPR in hospitals. Consequently, in most cases, ECPR was implemented within 60 minutes of the emergency call (the median time and interquartile range was 57 [48–71] minutes reported in the nationwide OHCA registry).^26^ On the other hand, the “scoop and run” strategy has faced some critique for losing the quality of resuscitation. Prehospital ECPR using a physician-staffed ambulance can resolve this issue. The ECPR team reaching the patients at the scene allows paramedics to continue high-quality resuscitation at the scene, and ECPR can be performed as soon as possible.

Another advantage of the prehospital ECPR system is its potential to improve accessibility for a larger number of people. Currently, in the US, less than 2% of patients with OHCA are available for ECPR due to geographical constraints and restricted accessibility.^29^ As mentioned above, minimizing the time required for ECPR is essential. Furthermore, providing ECPR requires a high level of expertise, well-trained teams, and sufficient medical resources because it has a substantial risk of severe complications.^27^ Accordingly, it may be better to perform ECPR in selected institutions with the appropriate capability to maintain quality.^28^ This limited accessibility is a challenge for establishing ECPR in a local system. To address this issue, prehospital ECPR in Utsunomiya is a good example of expanding geographical coverage and improving accessibility to ECPR for people living in the local community, as well as prehospital ECPR programs in other countries.^23^, ^30^

Several stakeholders must play important roles to establish this prehospital ECPR program as a local system. Citizens and paramedics generally initiate resuscitation. To seamlessly integrate ECPR strategies into prehospital settings and develop an efficient local system, effective collaboration with citizens, paramedics, and other stakeholders is crucial. Accordingly, the medical team holds events to highlight the importance of bystander CPR and introduces an innovative ECPR approach to the public. Furthermore, they routinely discuss their strategies in regular debriefing sessions and hold simulation training with paramedics to optimize their strategies. Prehospital ECPR is one of the many ECPR methods. As the saying goes, “If you’ve seen one EMS system, you’ve seen one EMS system,” We highlight that each region must assess its needs and collaboratively establish the most suitable system.

## The physician-staffed helicopter system in Japan

Next, we introduce the system of physician-staffed helicopters in Japan called “Doctor Heli.” The system dispatches a helicopter with an emergency physician and nurse upon the request of the dispatch center of the fire department and provides emergency medical care at the scene of the incident and during transfer to the hospital. This system plays a crucial role in non-urban regions of Japan, providing immediate emergency care for time-sensitive conditions. Japan's diverse geographical features, including its numerous mountains and islands, have led to certain areas facing challenges in accessing tertiary care in a timely manner. The helicopter can cover 50 km in 15 min, immediately provide an initial evaluation and resuscitation by an emergency physician and nurse over a wider area, and promptly transfer patients to a tertiary care center with resuscitation by the emergency medical team. Previously, it was reported that the physician-staffed helicopter system in Japan had some potential benefits over conventional EMS ground transport by ambulance, such as reduced mortality in patients with major trauma and shortened time to the angiography facility for patients with acute myocardial infarction or ischemic stroke.^32^, ^33^, ^34^, ^35^ In contrast, some observational studies in Japan indicated that compared to ground ambulances, the helicopter system was not associated with survival in patients with severe burns, isolated severe head trauma, or pediatric patients with severe head trauma.^36^, ^37^, ^38^ It may be because the helicopter system cannot provide an early definitive treatment to resolve time-critical conditions.

Furthermore, a novel activation system for physician-staffed helicopters (called “the D-Call Net system”) has recently been initiated in some areas of Japan. This D-Call Net system is implemented in some cars, and when a car is involved in an accident, it can automatically activate the physician-staffed helicopter system based on the probability of occupants sustaining severe injuries and information such as collision direction, velocity changes, and the activation status of seatbelts and airbags. This system can shorten the time to start resuscitation by 17 min compared with a conventional physician-staffed helicopter system.^39^ Between 2015 and 2023, the D-call net recorded 8,791 accident reports, resulting in 32 physician-staffed helicopter activities. Currently, D-Call Net has been implemented in approximately 4.2 million vehicles and linked to 53 physician-staffed helicopter systems in Japan^31^. The physician-staffed helicopter system collaborates with multiple disciplines such as local fire departments, hospitals, local governments, and industries to provide earlier dispatch and initiate prompt resuscitation.

## An example of the physician-staffed helicopter in non-urban areas

**Fig. 4:**
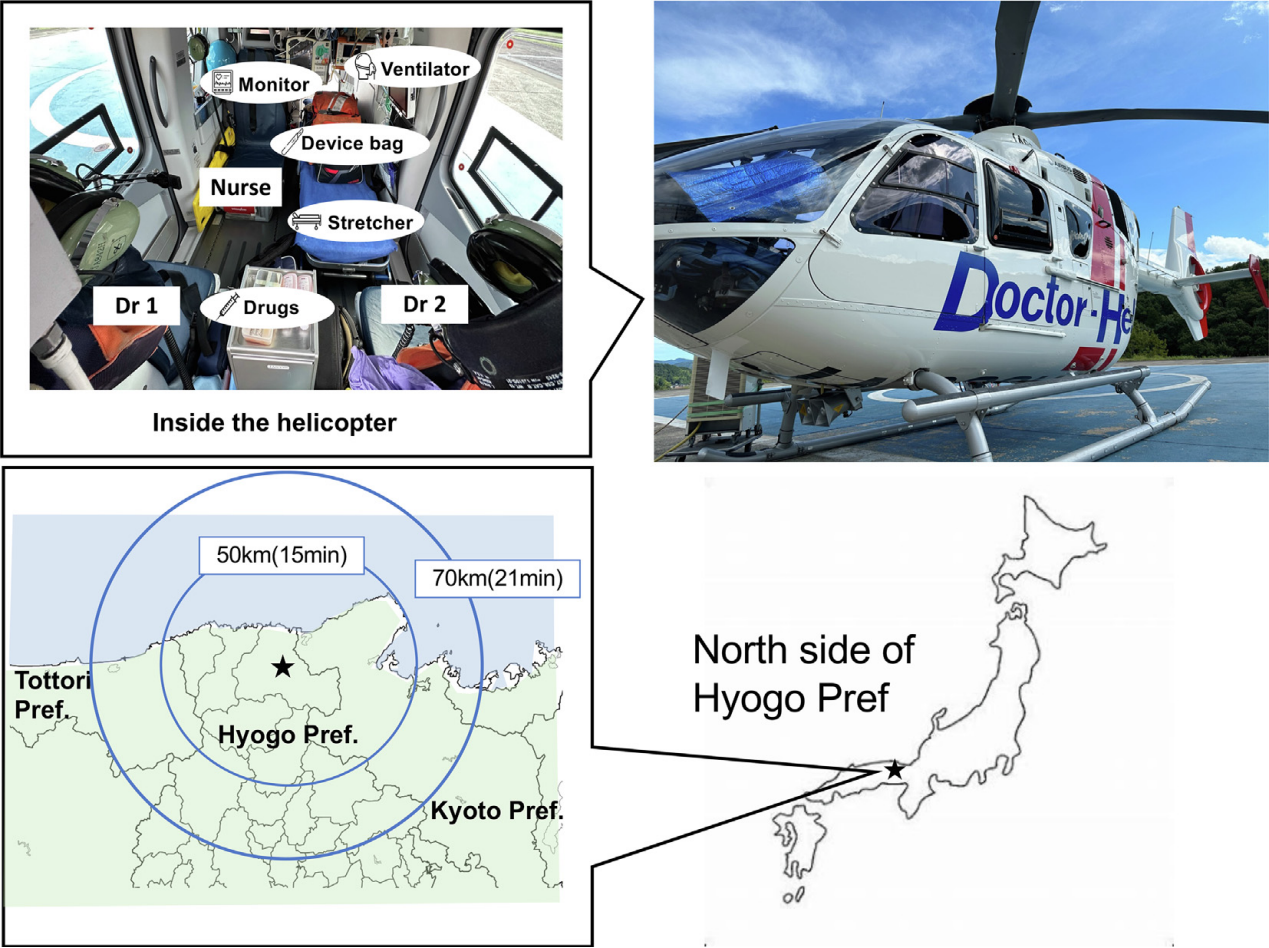
**Helicopter system of Northern part of Hyogo Prefecture.** The photo indicates the physician-staffed helicopter and the inside of the helicopter. Two physicians and one nurse work collaboratively to provide emergency care in the helicopter. The map shows the northern part of Hyogo Prefecture, highlighting the operational range of this physician-staffed helicopter. It is capable of covering a distance of up to 70 km within 21 minutes.

This section introduces more details about the physician-staffed helicopter system in non-urban areas and initiatives to improve the outcomes of out-of-hospital cardiac arrest patients using the example of the Tajima Emergency and Critical Care Medical Center (Fig. 4). This center is the only tertiary care hospital located on the north side of the Hyogo Prefecture, providing emergency care covering approximately 2,100 km^2^ (approximately 83% of this area is mountainous or forested), with a population of approximately 150,000 (of which 36.5% are 65 years or older).^40^ The primary challenge for the ambulance-based EMS system in this region is accessibility to tertiary care hospitals owing to geographical constraints. Typically, in this area, the ground transport of critically ill patients from an incident scene to a tertiary care hospital requires more than an hour. To address this problem, a physician-staffed helicopter system was developed in 2010. Since then, the Tajima Emergency and Critical Care Medical Center has provided physician-staffed helicopter emergency services every day unless weather conditions are not suitable. This physician-staffed helicopter system manages the highest number of cases annually in Japan (1,921 cases in 2022).^41^ The medical team boarding the physician-staffed helicopter included two doctors and one nurse trained in emergency care and disaster management at the scene, in addition to general emergency care.

Cardiac arrest is the most time-critical emergency condition, and the probability of obtaining favorable neurological outcomes and survival decreases over time. To address this issue, the following two strategies are emphasized in this physician-staffed helicopter system: The first was to minimize the time from emergency calls to hospital arrival. In this region, the dispatch center sets the “keywords” to suspect critically ill conditions such as “chest pain” or “sudden collapse,” and if the dispatcher detects the keywords in the call, they activate the physician-staffed helicopter.^42^ The dispatcher can also deploy the helicopter based on their clinical judgment apart from “keywords.” Further, paramedics contacting patients at the scene can also request the helicopter when they judge it is required; however, the dispatch system using the “keywords” can promote early recognition of time-critical conditions and send the helicopter before ambulances have arrived at the scene. Accordingly, 96% of the cases treated with the physician-staffed helicopter were activated as soon as the dispatch center received the call. This type of dispatch system shortened the time taken by emergency physicians.^42^ Furthermore, and the medical team boarding the helicopter also worked to shorten the time from arrival at the scene to departure from the scene. They prioritize departure to the hospital, minimize the time to initial assessment and stabilization, and perform time-consuming medical procedures such as obtaining an intra-venous or intra-osseous line, tracheal intubation, and point-of-care sonography during or after departing the helicopter. Previously, it was reported that securing intubation after a helicopter's take-off could safely reduce the duration of on-scene stay, as well as the time from dispatch to hospital arrival, compared to the policy of intubating before take-off [on-scene time, 7 vs. 14 min (*p* < 0.001), and the time from dispatch to hospital arrival was 33.5 vs 40.0 minutes (*p* < 0.001)].^43^

**Fig. 5:**
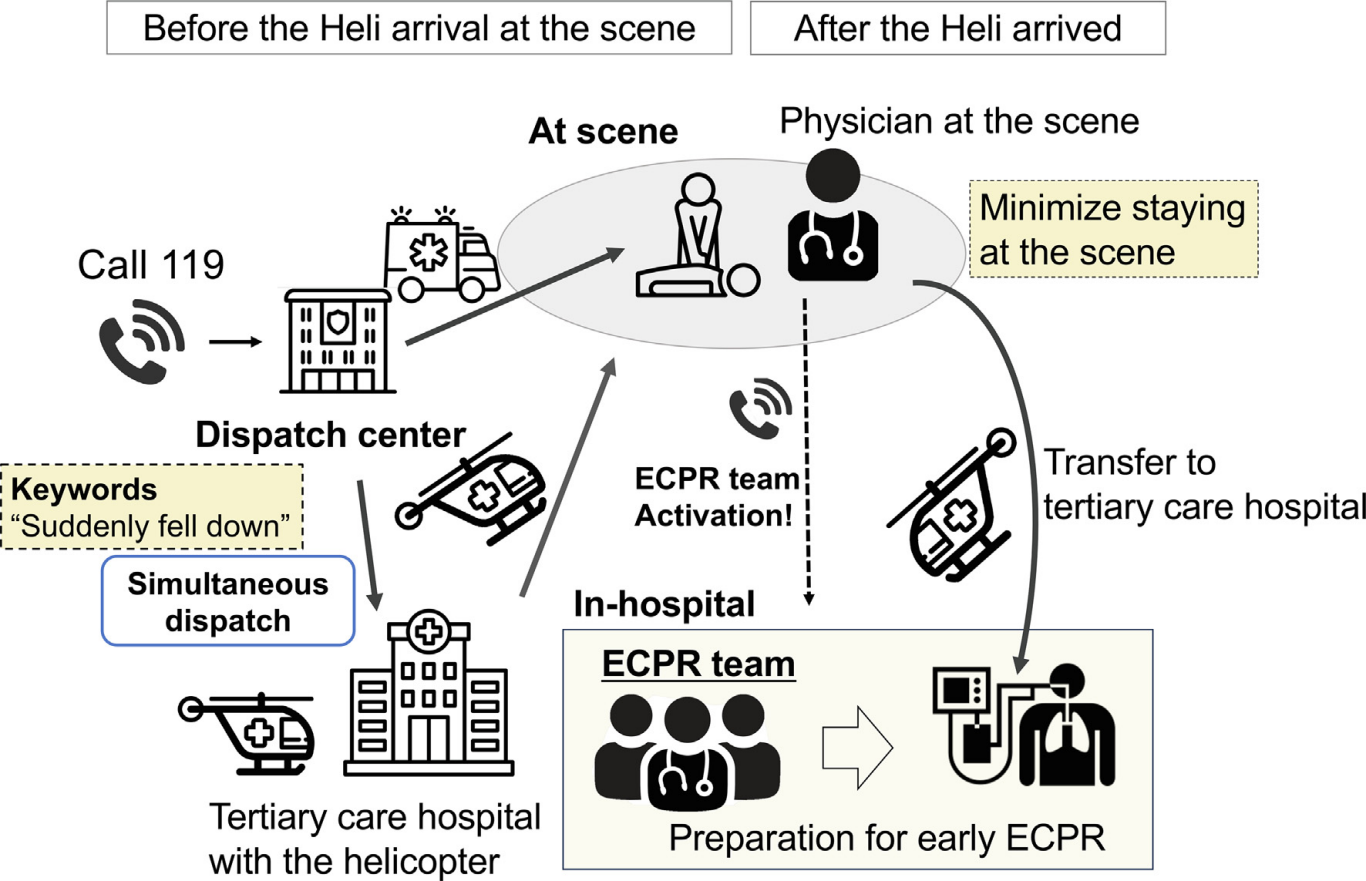
**The multidisciplinary approach for early ECPR implementation.** The “Keywords” dispatch system promotes the early dispatch of the physician-staffed helicopter to the scene. At the scene, the emergency physician activates the ECPR team in the hospital if they are suitable for ECPR while providing resuscitation. They minimize the time staying at the scene and bridge to ECPR as soon as possible. 119: Emergency call number for an ambulance in Japan, ECPR: Extracorporeal cardiopulmonary resuscitation.

Another strategy for minimizing the time to definitive treatment is to activate the team to provide definitive in-hospital treatment based on the judgment of the emergency physician boarding the helicopter. Especially for cardiac arrest cases, emergency physicians on the helicopter can judge the indication for ECPR by considering criteria such as initial cardiac rhythm, witness status, and activities of daily living before onset (Fig. 5). Then, they activate the ECPR protocol and the multidisciplinary team including the emergency physician, cardiologist, nurses, radiology department, angiography suite, and perfusionist to prepare for immediate implementation of ECPR before the patient arrives. After the helicopter lands, patients are directly transferred to the angiography suite to implement ECPR as soon as possible. Because the medical team on the helicopter includes two emergency physicians, one can continue to work on resuscitation while the other can make decisions comprehensively and communicate with the in-hospital team, leading to effective and efficient teamwork.

However, these strategies have limitations. The current dispatch system, which uses keywords to minimize undertriage and time to definitive treatment, has a high cancellation rate of 41% after dispatching the helicopters. Furthermore, most cardiac arrest cases (93.7%) had initial nonshockable rhythms, which were usually unsuitable for ECPR. Accordingly, the system requires sophisticated criteria or keywords to more accurately predict suitable patients during calls, improve efficiency, and maintain sustainability. Although this system must deal with these issues, physician-staffed helicopters provide high-quality resuscitation in non-urban and urban regions.

## Novel resuscitation system using hybrid ER

**Fig. 6:**
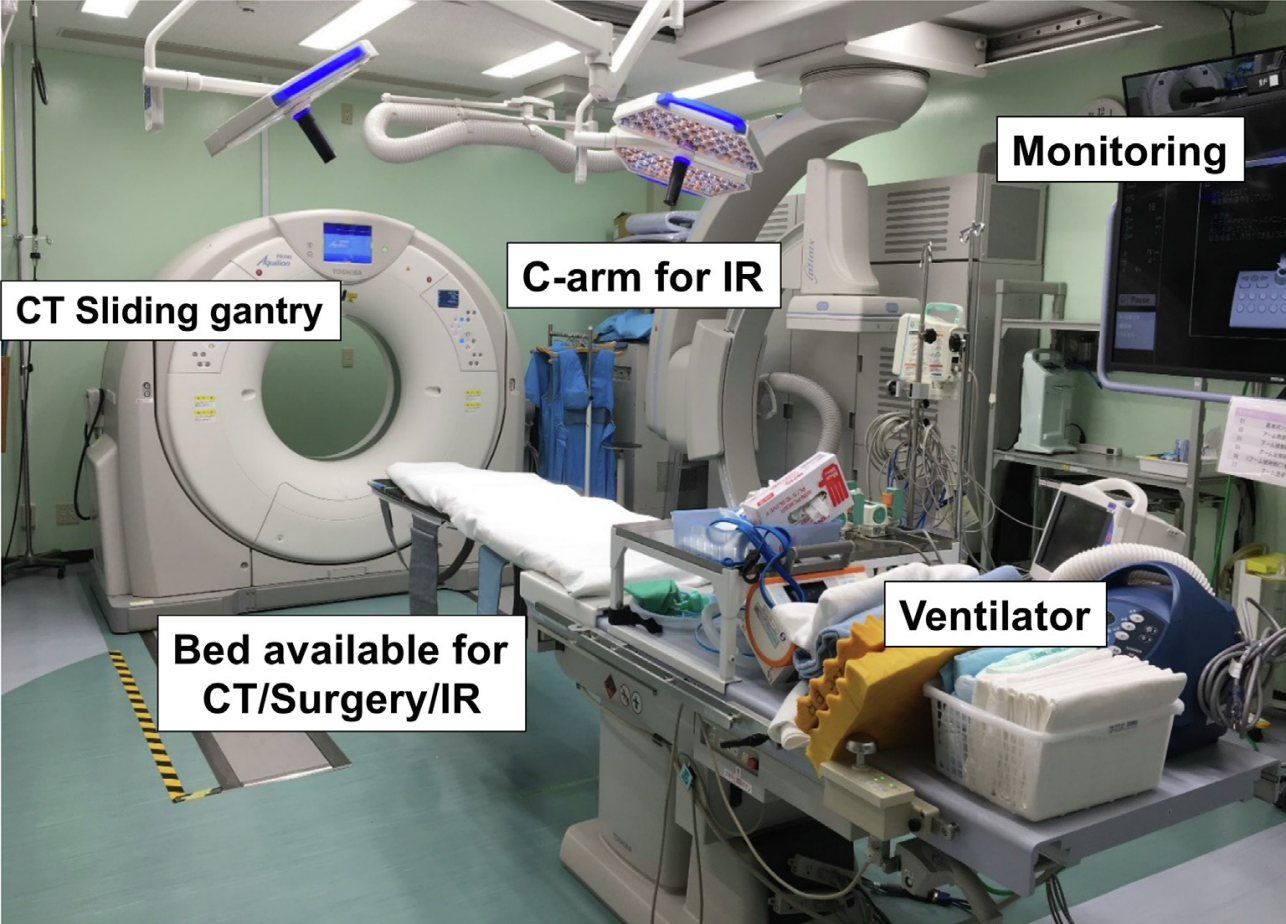
**Hybrid ER.** All life-saving procedures, including airway management, computed tomography (CT), emergency surgery, and interventional radiology (IR) can be performed promptly and safely on the same bed without transferring patients to other departments.

In addition, we introduce the novel resuscitation system using the hybrid emergency room (Hybrid ER). A hybrid ER system is defined as an integrated system that includes a resuscitation bay, emergency computed tomography (CT) room, interventional radiology (IVR) room, and operating room (Fig. 6).^14^, ^44^, ^45^, ^46^ When critically ill patients arrive at the hospital, they are immediately transferred from the ambulance to the hybrid ER. Diagnostic procedures, such as radiography and CT, were performed immediately, and catheter interventions or surgical procedures were initiated in the same place, if needed, without any transfer to other beds or facilities. This system has been highlighted for its benefits in managing patients with severe trauma and stroke to minimize the time to definitive therapy, such as hemostatic surgery, craniotomy for traumatic head injury, angioembolization for pelvic fracture, thrombolytic therapy, and endovascular treatment for ischemic stroke.^44^, ^47^, ^48^, ^49^

The hybrid ER also has great potential for cardiac arrest management, particularly for patients for whom ECPR is considered.^45^, ^50^ We identified two primary strengths in maximizing the effectiveness of the ECPR strategy. Because the hybrid ER facilitates fluoroscopy for angiography, it is expected to contribute to safe cannulation and shorten the time to implement ECPR. Previously, fluoroscopy-guided cannulation with sonography-supported puncture was reported to be associated with fewer complications during ECPR.^51^ The other single before-after study showed that before the implementation of the hybrid ER, the time to ECPR was typically over 40 min from hospital arrival; however, after implementing the hybrid ER, the mean time to start ECPR was within 25 min of arrival.^50^

Another feature of the hybrid ER is its innovative ECPR workflow. In this strategy, CT is performed immediately before ECPR initiation in patients suspected of having non-cardiac causes, such as OHCA patients with an initial non-shockable rhythm.^45^ During the scanning, mechanical CPR is continuously employed to avoid interruptions. To shorten the scanning time, head and chest CT scans were performed immediately without scanning scout images. This strategy enables clinicians to exclude irreversible causes that are generally unsuitable for ECPR, such as massive subarachnoid hemorrhage or acute aortic dissection, before initiating ECPR. A previous study investigating this strategy noted that the median time from hospital arrival to the end of CT was 170 seconds, and patients with OHCA due to irreversible causes were successfully detected before ECPR.^45^ This strategy, which is still being validated, could prevent unwarranted ECPR under irreversible conditions and aid in decision making.

Although hybrid ERs are promising, the hybrid ER itself do not save patients. A systematic approach with a trained multidisciplinary medical team, including emergency physicians, physicians with other specialties, nurses, and radiologists sharing treatment strategies. In particular, cooperation with other specialists (such as cardiologists, surgeons, and interventional radiologists) is essential to maximize the benefits of the hybrid ER system. The key to success is holding repeated meetings, discussing improvements continuously, and conducting numerous simulation training sessions to enhance a team’s proficiency. Additionally, as the adoption of hybrid ER broadens, usage strategies may become more complex. Therefore, standardizing management protocols, such as guidelines, specifically for hybrid ERs is required to establish a standardized system. Currently, only approximately 30 centers in Japan have hybrid ERs. However, we can anticipate further improvements in outcomes for critically ill patients, such as cardiac arrest patients, if the number of institutions with hybrid ER increases in the future.

## Conclusion

In this paper, we provide an overview of the novel and innovative resuscitation systems in Japan. Although these revolutionary approaches may improve the outcomes of patients with OHCA, evidence of their effectiveness remains limited. In addition, it is crucial to ensure cost-effectiveness and sustainability. We will continue to work diligently to assess the effectiveness of these systems and develop cost-effective and sustainable systems.

## CRediT authorship contribution statement

**Yohei Okada:** . **Kensuke Fujita:** Writing – review & editing, Writing – original draft, Conceptualization. **Takayuki Ogura:** Writing – review & editing. **Tomokazu Motomura:** Writing – review & editing. **Yuita Fukuyama:** Writing – review & editing, Writing – original draft. **Yuki Banshotani:** Writing – review & editing, Writing – original draft. **Rina Tokuda:** Writing – original draft, Conceptualization. **Shinichi Ijuin:** Writing – original draft, Conceptualization. **Akihiko Inoue:** Writing – review & editing, Conceptualization. **Haruka Takahashi:** Visualization. **Shoji Yokobori conceptualized:** Writing – review & editing, Writing – original draft, Conceptualization.

## Declaration of competing interest

The authors declare the following financial interests/personal relationships which may be considered as potential competing interests: ‘YO received a research grant from the ZOLL Foundation and overseas scholarships from the Japan Society for the Promotion of Science, FUKUDA Foundation for Medical Technology, and the International Medical Research Foundation. These organizations had no role in the writing of this review’.
